# Age cutoff in lymphoma diagnosis

**DOI:** 10.18632/aging.100774

**Published:** 2015-07-06

**Authors:** Chi Y. Ok, Ken H. Young

**Affiliations:** The University of Texas MD Anderson Cancer Center, Department of Hematopathology, Houston, TX 77230, USA

Aging is characterized by immunosenescence, decreased immune response and dysregulated coordination between inflammatory and inflammation-neutralizing processes. The decline in immune competence is invariably seen with aging. As a consequence, viral or bacterial infections, chronic inflammatory disorders, autoimmune diseases, and cancers are more prevalent in elderly. In addition to genomic and epigenomic instability with age, decreased immunosurveillance plays an importance role in tumorigenesis. In a young individual, there are strong supplies of cytokines including insulin-like growth factor-1, keratinocyte growth factor and interleukin-7 that induce production of naïve T-cells with a diverse T-cell receptor repertoire. When antigen is presented by antigen-presenting cells, effector T-cells and memory T-cells are quickly produced. In an aged person, there is a significant decline in the stimulatory cytokines, hence leading to a decreased T-cell population. Often, chronically stimulated effector T-cells and memory T-cells with limited repertoire diversity predominate and progressive expansion of oligoclonal T-cells, particularly CD8^+^, CD27^−^, CD28^−^, CD45RA^+^ and CD57^+^ T-cells are seen [1]. Analysis of immuno-globulin heavy variable gene (IGHV) complementarity determining region (CDR) 3 using blood mononuclear cells showed that B-cell receptor repertories dramatically decline with age in parallel with clonal expansion of B-cells *in vivo*. Imbalance between inflammatory and inflammation-neutralizing processes prompts a low-grade inflammatory process. Chronic inflammation induces radical oxygen species (ROS) that can cause dysregulation of critical signaling pathways such as p53, retinoblastoma (Rb), nuclear factor (NF)-κB, and mitogen activated protein kinases (MAPKs).

Diffuse large B-cell lymphoma (DLBCL) is the most common type of lymphoma and encompasses a heterogeneous group of tumors with several variants, subgroups, subtypes and entities. Gene expression profiling has identified three distinct groups of DLBCL – germinal center B-cell-like (GCB), activated B-cell-like (ABC), and mediastinal large B-cell lymphoma (MLBCL). Elderly patients commonly have ABC subtype of DLBCL with frequent BCL2 expression and genetic complexity. Chromosomal gains in 1q21, 18q21, 7p22, 7q21 and 3q27 are more often seen with age, but IRF4 break is less frequent in elderly. Epidemiological data shows that there is a significant increase of DLBCL incidence with age. The epidemiological association with DLBCL and aging is partially explained by data from Jaiswal and colleagues [2]. Analysis of whole-exome sequencing data from DNA in the peripheral blood cells of 17,182 persons without hematologic malignancies showed that prevalence of somatic mutations increase with age and the cumulative incidence of hematologic cancer is quantitatively associated with presence of somatic mutations [2]. Epigenetic alterations are also associated with development of cancer. Genome-wide alteration in methylation is extensively found with age. A model of aging epigenome postulates that stochastic errors in DNA methylation maintenance during DNA replication are accumulated with aging, causing epigenetic mosaicism with restricted differentiation in affected stem cells. Unaffected stem cells acquire selective growth advantage with increased possibility of clonal expansion [3]. A role of epigenetics in DLBCL lymphomagenesis is indirectly seen with the fact that mutations in *MLL2*, which encodes a histone methyltransferases, and *MEF2B*, which encodes a DNA-binding protein in cooperation with CREBBP and EP300 in histone acetylation, are detected in approximately 30% and 10% patients, respectively. In comparison, younger or pediatric DLBCLs are rare and comprise approximately 5% of the patients. They are often of GCB subtype with genetic abnormalities involving only a few signaling pathways, suggesting different biological signature and genomic/epigenomic mechanism in the lymphoma development.

Epstein-Barr virus (EBV)-positive DLBCL of the elderly is a molecularly distinct variant of DLBCL, defined as a monoclonal B-cell proliferation that occurs in patients >50 years of age without a history or clinicopathologic evidence of immunodeficiency [4]. It is characterized by enhanced activity of the NF-κB, signal transducer and activator of transcription 3 (STAT3), MEK/ERK and phosphoinositide 3-kinase (PI3K)/Akt pathways, mostly induced by EBV-derived signals. It has been believed that EBV+ DLBCL of the elderly might be caused by the senescence of the immune system as a part of the normal aging because of its similarity to immunodeficiency-associated lymphoproliferative disorders except overt immunodeficient state. The sharing common features include similar EBV latency pattern, morphologic similarities and presence of monoclonal T-cell populations. Prevalence of EBV positivity in DLBCL increases with age, up to 30% in patients >90 years. Although a trend that EBV positivity is associated with increased age is observed, age cutoff (>50 years) in definition in 2008 WHO lymphoma classification seems to be arbitrary. Well-documented cases of DLBCL with EBV infection in immunocompetent adults ≤50 years have been reported, questioning the rational of the age cutoff used in the current clinical practice [5,6]. Our recent study showed that EBV+ DLBCL in patients >50 years are clinically and biologically similar to those ≤50 years in clinicopathologic features, immunophenotypes, gene expression profiling, microRNA profiling and treatment outcome [7]. Immunosenescence might be an underlying predisposing factor of lymphomagenesis in EBV+ DLBCL of the elderly, but lack of difference in multiple parameters between patients >50 years and ≤50 years prevents us from using the current age cutoff in lymphoma definition and classification. We recommend that the entity be defined as EBV^+^ DLBCL and regarded as a biologically distinct variant of DLBCL. Its genomic or epigenomic aberrations and immune-regulatory defects distinguish from EBV^−^ *de novo* DLBCL, suggesting that these patients may benefit immunotherapy, alone and in combination, with targeted therapeutic interventions.

**Figure 1:**
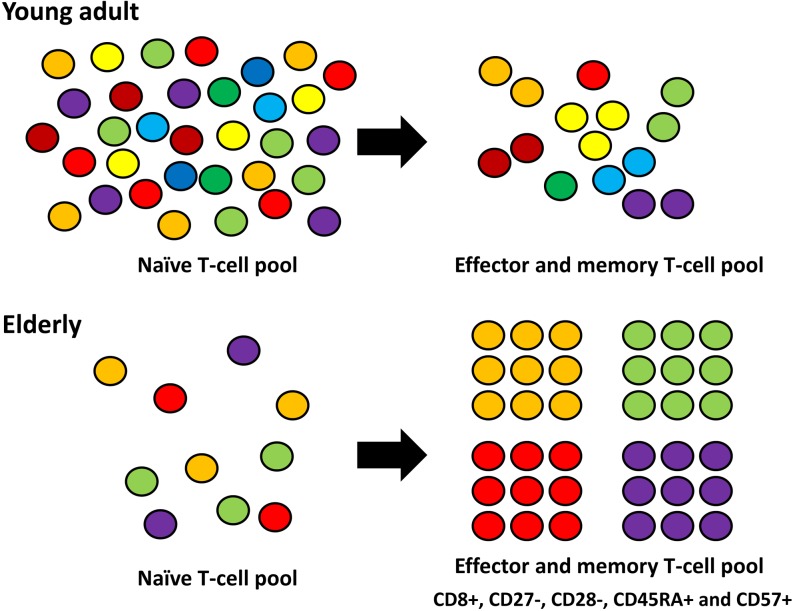
Compared with an elderly, the absolute number of naïve T-cells is higher with diverse T-cell receptor repertoire in a young adult. When antigen is presented by antigen presenting cells, effector T-cells and memory T-cells are quickly produced. In an elderly, the absolute number of naïve T-cells are decreased with limited T-cell receptor repertoire. Chronically stimulated effector T-cells and memory T-cells predominate with progressive expansion of oligoclonal T-cells.

## References

[1] Ouyang Q (2003). Mech Ageing Dev.

[2] Jaiswal S (2014). N Engl J Med.

[3] Issa JP (2014). J Clin Invest.

[4] Young KH (2014). Neoplastic Hematopathology.

[5] Ok CY (2014). Clin Cancer Res.

[6] Ok CY (2013). Blood.

[7] Ok CY (2015). Oncotarget.

